# Neutron reflectometry on highly absorbing films and its application to ^10^B_4_C-based neutron detectors

**DOI:** 10.1098/rspa.2015.0711

**Published:** 2016-01

**Authors:** F. Piscitelli, A. Khaplanov, A. Devishvili, S. Schmidt, C. Höglund, J. Birch, A. J. C. Dennison, P. Gutfreund, R. Hall-Wilton, P. Van Esch

**Affiliations:** 1 European Spallation Source ERIC, PO Box 176, Lund 22100, Sweden; 2 Institut Laue-Langevin (ILL), 71, Avenue des Martyrs, Grenoble 38042, France; 3 Department of Physics, University of Perugia, Piazza Università 1, Perugia 06123, Italy; 4 Ruhr-Universität Bochum, Bochum 44780, Germany; 5 Thin Film Physics Division, Linköping University, Linköping 58183, Sweden; 6 Department of Physics and Astronomy, Uppsala University, BP 516, Uppsala 75120, Sweden; 7 Mid-Sweden University, Sundsvall 85170, Sweden

**Keywords:** neutron-induced fluorescence, neutron reflectometry, boron-10, neutron detection

## Abstract

Neutron reflectometry is a powerful tool used for studies of surfaces and interfaces. The absorption in the typical studied materials is neglected and this technique is limited only to the reflectivity measurement. For strongly absorbing nuclei, the absorption can be directly measured by using the neutron-induced fluorescence technique which exploits the prompt particle emission of absorbing isotopes. This technique is emerging from soft matter and biology where highly absorbing nuclei, in very small quantities, are used as a label for buried layers. Nowadays, the importance of absorbing layers is rapidly increasing, partially because of their application in neutron detection; a field that has become more active also due to the ^3^*He*-shortage. We extend the neutron-induced fluorescence technique to the study of layers of highly absorbing materials, in particular ^10^B_4_C. The theory of neutron reflectometry is a commonly studied topic; however, when a strong absorption is present the subtle relationship between the reflection and the absorption of neutrons is not widely known. The theory for a general stack of absorbing layers has been developed and compared to measurements. We also report on the requirements that a ^10^B_4_C layer must fulfil in order to be employed as a converter in neutron detection.

## 1. Introduction

Neutron reflectometry is a powerful tool used for studies of surface chemistry, surface magnetism and solid films [1]. The reflection of neutrons was first demonstrated by Fermi and Zinn in 1944 [2]. In most cases, the absorption in the media can be neglected and the total number of neutrons remains constant via the sum rule

1.1 1−R−T=0,

where *R* and *T* are reflectivity and transmittance, respectively, normalized to the incident number of neutrons. Therefore, most interfacial investigations are limited only to the measurement of the reflectivity. Measuring the transmittance in some cases can provide extra information about absorption or other anomalous scattering in the sample. However, the measurement of transmittance is often complicated due to refraction of transmitted neutrons and secondary scattering/absorption in the sample holder.

The absorption on the other hand can be in some cases estimated by measuring the prompt *α* or *γ* particle response, i.e. the neutron-induced fluorescence. The following isotopes are suitable for such investigations: ^3^He, ^6^Li, ^10^B, ^149^Sm, ^151^Eu, ^156^Hg, ^155^Gd and ^157^Gd. Several studies have been realized through (*n*,*α*) reaction on ^6^Li [3] or (*n*,*γ*) reaction on ^155^Gd and ^157^Gd [4]. The primary interest in this technique emerges from the fields of soft condensed matter physics and biology where the use of labelled molecules can allow enhanced sensitivity to absorption of low concentrations while also providing the structural information normally associated with neutron reflectometry [5]. In these studies, only a small fraction of the sample is composed of absorbing nuclei and the information on the buried labelled molecules is given by the neutron-induced fluorescence below the critical edge (or alternatively called critical angle). The latter is given by the condition that the normal component of the energy of the impinging neutron equals the barrier potential of the interface. The critical angle for total reflection is such that the reflectivity of neutrons of a given wavelength from a bulk interface is unity at lower glancing angles (ignoring absorption effects) and falls sharply at larger angles [1].

The simultaneous measurement of the reflectivity and absorption has a great advantage with respect to the classical measurement of reflectivity only; the two sets of correlated data make this technique more sensitive to single chemical elements. Despite the development of isotopic substitution, classical scattering techniques are intrinsically lacking direct chemical sensitivity. That is, in case of interfaces with graded scattering length density (SLD) profiles, the distributions of different molecules cannot be determined unambiguously [5].

In this manuscript, we extend the neutron-induced fluorescence technique to the study of dense layers of highly absorbing materials, in particular on ^10^B_4_C layers. Specifically, the number density of the absorbing media can be determined by measuring reflectivity and absorption simultaneously. The two sets of data have been fitted using a set of equations we derived from the theory we developed for generic stack of a highly absorbing layers. The theory of neutron reflectometry is a commonly studied topic; however, the subtle relationship between the reflection and the absorption of neutrons is not widely known, in particular when a strong absorption is present.

Nowadays, the importance of ^10^B_4_C layers is increasing. Most of the neutron sources in the world, such as the European Spallation Source (ESS) [6,7] in Sweden, are necessarily pushing the development of their detector technologies, due to the increased flux available for the neutron scattering science and the scarcity of ^3^He, the so-called ‘Helium-3 crisis’ [8,9]. ^10^B along with ^3^He and ^6^Li isotopes are the main actors in thermal neutron detection due to their large absorption cross sections [10]. Concerning small-area detectors (less than 1 m^2^), the current detector technology is reaching fundamental limits in position resolution and rate capability. The Multi-Blade [11–14], the Jalousie detector [15,16], the A1CLD [17] and many others [18,19] are an example of the detector developments for small area coverage which exploit solid neutron converters operated at a grazing angle (between 0° and 10°) in order to increase the neutron detection efficiency. For detection applications, neutron reflection from the detector elements must be avoided because it limits the maximum efficiency that can be attained and it may give rise to misaddressed events.

Recently, high-quality, low-cost production of square metres of ^10^B_4_C [17,20] became possible and some of the detector developments are focused on the application of such films in inclined geometry.

Neutron reflectometry with neutron-induced fluorescence is a powerful tool to investigate the performance of highly absorbing layers employed in neutron detection. In this manuscript, we also report on the requirements that a converter layer must fulfil to be employed in a detector to avoid reflection. We characterized the ^10^B_4_C layer when deposited on both Si and Al substrates.

This manuscript is composed of three parts: the theoretical description of the reflectivity with absorption, the simultaneous measurement of them on ^10^B_4_C layers and the requirements for converter coatings in thermal neutron detection. The theory has been described in detail in the first part in order to have a comprehensive model that has been used in the experimental part of this manuscript to fit the data simultaneously. Once the chemical composition of the layer has been determined also thanks to the higher sensitivity of this technique to single isotopes such as ^10^B in our layer, the requirements for such a layer to be employed in a neutron detector are given in the concluding section of this paper. Not only the amount of ^10^B in the layer is relevant, this being the isotope used in neutron detection, but also the reflectivity of the surface of layer if employed at a grazing angle in a neutron detector.

### (a) Theory of neutron reflection on highly absorbing layers

The reflection of neutrons from surfaces is a phenomenon caused by the change of refractive index across the interface analogous to that for light. The analysis of specular neutron reflectivity reveals the nuclear density profile perpendicular to the surfaces and interfaces. The sensitivity of neutron reflectivity to interfaces is due to the fact that the kinetic energy of a neutron projected on the surface normal at grazing incidence is comparable with the potential energy of the reflecting interface, *V* . At the same time, the wavelength corresponding to this component of the kinetic energy matches often the thickness of thin films of interest well.

When dealing with neutron absorbers, the theory describing the physical process of reflection has to be modified in order to not only take the possibility for a neutron to be scattered into account, but also its absorption by nuclei. The sum rule in this case will take the following form:

1.2 1−R−T−A=0,

where *R*, *T* and *A* are, respectively, reflectivity, transmittance and absorption normalized to the incident number of neutrons. The scattering length of a nucleus is a complex quantity. Its real and imaginary parts can be associated with the scattering process but only its imaginary part to the absorption [21,22].

The scalar potential *V* in the Schrödinger equation, $-(\hbar^{2}/2m_{n})\nabla^{2}\Psi +V\Psi =E\Psi$, will contain the contribution given by the absorption, and it is

1.3 $$V=\frac{2\pi \hbar^{2}}{m_{n}}(N_{b}^{\mathrm{real}}+iN_{b}^{\mathrm{im}}),$$

where *m*_*n*_ is the neutron mass and where the SLD *N*_*b*_ can be calculated according to $N_{b}=\sum_{i}b_{i}n_{i}$ (with *b*_*i*_ the scattering length of the *i*th species, isotope and *n*_*i*_ its number density). The solutions of the Schrödinger equation, with a complex potential, can still be written as for a real potential [1]; however, the wavevectors will be complex quantities. The solutions of the Schrödinger equation can be still factorized as two plane waves, one orthogonal to the surface and one parallel. The complex potential the neutrons experience affects only the normal component of the momentum. Continuity of wave functions and their derivatives at boundaries lead to the unaltered conservation of the parallel wavevector. Thus, we refer only to the normal component of the wave functions and we denote the wavevector in a generic layer *n* simply as *k*_*n*⊥_=*k*_*n*_.

The reflectivity profile can be measured in time-of-flight (ToF) or in monochromatic mode without affecting the results (if we can assume constant imaginary SLD, meaning there are no absorption resonances). By denoting with *k*_0_ the normal component of the incoming wavevector, the reflectivity only depends on *θ* and λ through $k_{0}=q/2=(2\pi /\lambda )\sin(\theta )$. Thus any method (ToF or monochromatic) is used to get a value for the measured reflectivity the result is the same for same *q*.

The change in the normal wavevector has an imaginary part given by the complex potential that results in an exponentially reduced amplitude (with the imaginary part of the wavevector in the media) of the wave function [22]. As such, the absorption has been taken into account in the amplitudes of the wave functions. An equivalent way of looking at the absorption is as a negative source term that appears in the continuity equation. This gives an indication of where the absorption, which is included in the wave functions, takes place. If there would not have been any imaginary potential, probability is conserved in quantum mechanics. There are no source terms in the continuity equation if the potential is purely real. The source term is then entirely related to the imaginary part of the potential. The continuity equation gives the normalization of the wave functions; in the region where the material is an absorber it has to be generalized considering the probability for a neutron to be absorbed [23]. The continuity equation can be written as

1.4 ∂P(r¯,t)∂t+∇⋅J(r¯,t)=−2ℏP(r¯,t)Im{V} ⟹ ∇⋅J(r¯,t)=−4πℏmnP(r¯,t)Nbim,

where $P(\bar{r},t)$ and $J(\bar{r},t)$ are the probability density function and the probability current, respectively; assuming stationary conditions: $\partial P(\bar{r},t)/\partial t=0$.

We can use equation (1.4) to determine the amount of absorption in a certain volume, for instance, the entire layer of absorbing material with thickness *d*. Mathematical consistency between equations (1.2) and (1.4) then requires that the following relation holds:

1.5 $$A=\frac{m_{n}}{\hbar k_{0}}\int_{0}^{d}\nabla \cdot J(z,t)\;dz=-\frac{4\pi }{k_{0}}\int_{0}^{d}P(z,t)N_{b}^{\mathrm{im}}\;dz=-\frac{4\pi }{k_{0}}\int_{0}^{d}|Y_{z}{|}^{2}N_{b}^{\mathrm{im}}\;dz,$$

where *k*_0_ the normal component of the incoming wavevector, *d* is the thickness of the absorbing layer, $N_{b}^{\mathrm{im}}$ is the imaginary part of the SLD of the absorbing medium and *Y*_*z*_ is the solution of the Schrödinger equation in the absorbing layer. The solutions of the Schrödinger equation in the different regions can be calculated iteratively according to the Parratt formalism [24].

**Figure 1.**
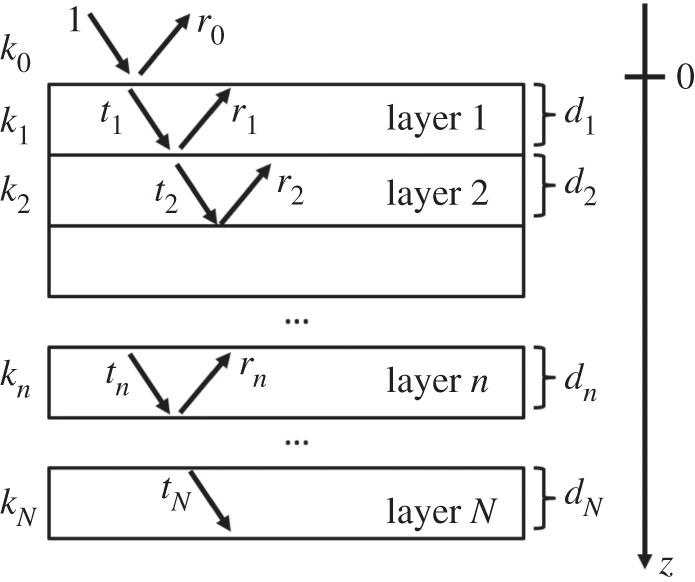
A stack of *N* layers of thicknesses *d*_*n*_; *k*_*n*_ is the normal component of the wavevector, *r*_*n*_ and *t*_*n*_ are the complex amplitudes of the wave functions in the *n*th layer.

In order to generalize to a multi-layer system, let us consider a stack of *N* layers of thicknesses *d*_*n*_; *k*_*n*_ is the normal component of the wavevector in each region, *r*_*n*_ and *t*_*n*_ are the complex amplitudes of the wave functions in the *n*th layer (figure 1). In the first medium (generally air), the incoming amplitude is 1 and the reflected amplitude is *r*_0_. We consider the amplitude in the layer *N* to be only transmitted since we assume it to be a substrate of infinite thickness. In the generic region, *n* the normal component of the solution of the Schrödinger equation is

1.6 $$Y_{n}(z)=t_{n}\;e^{+ik_{n}z}+r_{n}\;e^{-ik_{n}z}.$$

Note that not all the layers must necessarily be an absorber. With absorber we mean here a material which has an absorption cross section that cannot be neglected with respect to the scattering cross section (*σ*_*a*_≈*σ*_*s*_). For most materials the absorption cross section is a few orders of magnitudes smaller than the scattering cross section. The total absorption is given by the sum of the contributions of the single layers. Equation (1.5) can be used to calculate the absorption of each layer considering the solution *Y*_*n*_ in the region of thickness *d*_*n*_ and the respective imaginary part of the SLD $N_{b}^{\mathrm{im}}$. Let us consider a finite thickness as in [4], *d*, of absorbing material deposited on a substrate, e.g. Si. In this specific case, we consider *N*=2. We identify three regions delimited by two interfaces: air/absorber (*z*=0) and absorber/substrate (*z*=*d*). We denote *k*_*n*⊥_=*k*_*n*_ with *n*=0,1,2, the normal component of the wavevectors in the three regions is defined by the potentials *V*_*n*_.

The reflection and transmission probabilities are given by

1.7 $$R=\frac{|J_{r}|}{|J_{i}|}\;\mathrm{and}\;T=\frac{|J_{t}|}{|J_{i}|}$$

with

1.8 $$|J_{i}|=\frac{\hbar \;k_{0}}{m_{n}},\;|J_{r}|=\frac{\hbar \;k_{0}}{m_{n}}(r_{0}\cdot r_{0}^{*})\;\mathrm{and}\;|J_{t}|=\frac{\hbar \;k_{2}}{m_{n}}(t_{2}\cdot t_{2}^{*}),$$

where *J*_i_, *J*_r_ and *J*_t_ are the probability current of the incoming, reflected and transmitted waves, respectively. The measured reflectivity, the transmission inside the substrate and the absorption in the layer are

1.9 $$\begin{array}{rl} R & =r_{0}\cdot r_{0}^{*}\\ T & =\frac{k_{2}}{k_{0}}(t_{2}\cdot t_{2}^{*})\\ \mathrm{and}\;\;A & =1-R-T=\frac{1}{k_{0}}\int_{0}^{d}\nabla \cdot J_{1}(z,t)\;dz=-\frac{4\pi }{k_{0}}\int_{0}^{d}|Y_{z}{|}^{2}N_{b}^{\mathrm{im}}\;dz, \end{array}\}$$

where *J*_1_ is the probability current calculated for *Y*_*z*_, and *r*_0_, *t*_2_ are the amplitudes of the waves in the first and third medium. The three different expressions in the third line of equation (1.9) are three mathematically equivalent ways to calculate the absorption that results from the introduction of an imaginary part to the potential.

**Figure 2.**
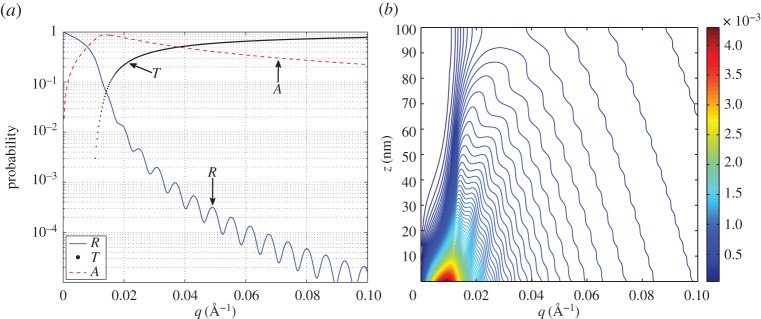
The calculated reflectivity (*R*), transmission (*T*) and absorption (*A*) for a 100 nm ^10^B_4_C layer deposited on Si (*a*), the probability for a neutron to be absorbed in the layer as a function of *z* and *q*, i.e. the quantity $-(4\pi /k_{0})|Y_{z}{|}^{2}N_{b}^{\mathrm{im}}$ (*b*); the colour scale is expressed in inverse ångström. (Online version in colour.)

As example we take a ^10^B_4_C layer (*N*_*b*_=(2.5−1⋅*i*)×10^−6^ Å^−2^) of *d*=100 nm deposited on Si (*N*_*b*_=2.14×10^−6^ Å^−2^). In figure 2*a*, we show reflectivity, transmission and absorption, as calculated in equation (1.9) as a function of *q*. In figure 2*b*, we show the probability for a neutron, carrying a given *q*, to be absorbed at certain depth in the layer, i.e. the quantity $-(4\pi /k_{0})|Y_{z}{|}^{2}N_{b}^{\mathrm{im}}$. We note that absorption increases in proximity of the critical edge (*q*_c_) that is, in this case, at about *q*_c_=0.01 Å^−1^.

## 2. Experimental set-up

### (a) Preliminary characterization of the ^10^B_4_C-layers

^10^B_4_C thin films were deposited in Linköping University by the Thin Film Physics Division in an industrial deposition chamber (CemeCon AG, Germany) using direct current magnetron sputtering (DCMS). The films were synthesized from enriched ^10^B_4_C sputter targets in an Ar discharge. The sputter targets were enriched to a ^10^B content of about 98% (specified by the supplier) of the total boron content [20,25,26].

A thin layer of about 100 nm ^10^B_4_C film was deposited on Si(001). A thick layer of about 1 μm was deposited on Si(001) and aluminium (EN AW-5083) substrates, the latter is a common substrate used in neutron detectors [12,27].

The chemical composition of the films were investigated by X-ray photoelectron spectroscopy (Axis UltraDLD, Kratos Analytical, UK) using monochromatic X-ray radiation (*h*_*ν*_=1486.6 *eV*). The base pressure in the analysis chamber during acquisition was less than 10^−7^ Pa. X-ray photoelectron spectroscopy (XPS) is a surface-sensitive technique; data contain information of the material surface and sub-surface layers of approximately 10 nm in depth. The XPS core-level spectra of the B1s, Ar2p, C1s and O1s regions were recorded on the as-received sample and after each Ar^+^ etching step during depth profiling. Depth profiling was performed with an Ar^+^ ion beam, rastered over an area of 3×3 mm^2^ at an incidence angle of 20°. The Ar^+^ etch sequence removed 5 nm of material in each of the first two steps, corresponding to a sputter etch time of 85 *s* per step. The following steps removed each 10 nm of ^10^B_4_C corresponding to a sputter etch time of 171 *s*. The composition of the 100 nm thin film was extracted after each Ar^+^ etch step. Here, the corresponding core-level spectra were evaluated after subtracting a Shirley-type background using the CasaXPS software and elemental cross sections provided by Kratos Analytical. After removing the first 10 nm of oxidized film surface, the composition in the bulk of the film did not vary by more than 1 %. Table 1 shows the composition of the ^10^B_4_C layer as a function of the sampling depth. It shows that there is a thin (≈5 nm) partially oxidized layer on the surface of the ^10^B_4_C layer. In order to accesses the surface roughness of the ^10^B_4_C film, atomic force microscope (AFM) scans were performed in tapping mode using a Digital instruments Multimode equipped with a silicon tip (type: PPP-NCHR, Nanosensors, Swizerland) with a resonance frequency of 284 kHz. Four measurements with a scan size 1×1 μm on different sample locations were performed. The roughness of the 100 nm thick ^10^B_4_C film on Si when excluding surface particles was extracted to be (0.47±0.02) nm. The roughness when including the surface particles was (6.12±0.02) nm.

**Table 1.** ^10^B_4_C film composition with progressing film depth from the surface as obtained from relevant XPS core-level spectra. The error on the composition never exceeds ±2%. As explained in the text, the ^11^B percentage is kept fixed.

depth (nm)	^10^B (*At*.%)	^11^B (*At*.%)	C (*At*.%)	O (*At*.%)
0	38.2	1.5	41.5	18.8
5	75.8	1.5	19.6	3.1
≥10	77.3	1.5	19.3	1.9

X-ray reflectivity (XRR) was performed to determine the layer density using a Philips X'Pert Pro MRD diffractometer equipped with a hybrid mirror monochromator and a 2-bounce Ge 220 triple-axis crystal analyser. The film density was determined to be (2.45±0.05) g cm^−3^ by fitting the XRR data using a two layer model with the X'pert reflectivity software.

For the analysis of the reflectometry and fluorescence data, we assume the amount of ^11^B fixed at 1.5%. This assumption is made to reduce the number of free parameters. Moreover, ^11^B and *C* are not distinguishable in neutron reflectometry due to their similar scattering lengths [21], hence any percentage we fix of ^11^B can be considered as a part of the total *C* amount.

According to the composition (table 1) and the mass density, we expect the scattering length density $N_{b}=\sum_{i}b_{i}n_{i}$ of the layers to vary in the range given in table 2.

**Table 2.** Scattering length density (*N*_*b*_) of the sputtered ^10^B_4_C layer calculated according to the depth and the composition given in table 1. The minimum and the maximum values are calculated according to the range of the layer mass density.

depth (nm)	$\min N_{b}$ (10^−6^ Å^−2^)	$\max N_{b}$ (10^−6^ Å^−2^)
0	4.72−0.49 *i*	4.92−0.51 *i*
5	2.06−1.10 *i*	2.14−1.14 *i*
≥10	1.95−1.13 *i*	2.03−1.17 *i*

Note that all the elements in the film, including ^10^B, contribute to the real part of *N*_*b*_ and almost only the ^10^B amount determines its imaginary part. In fact the imaginary part of the ^10^B scattering length is about six order of magnitudes larger of that of any of the other components [21].

### (b) Reflectivity and absorption measurements

Two experiments have been performed. The first set of data was taken using D17 [28] at ILL which was used as a ToF reflectometer to preliminary quantify the actual reflectivity of the coatings and to have a direct measurement of the reflection as a function of the neutron wavelength for different angles. A second experiment has been performed on SuperADAM [29] at ILL, which is a monochromatic reflectometer in a set-up that also included a *γ*-ray spectrometer. The two experiments allow the comparison of the two techniques (ToF and monochromatic), and additionally provide information on neutron converter reflectivity and layer composition.

The 1 μm thick ^10^B_4_C layers deposited on both Si and Al were measured on D17. Both 1 μm and 100 nm layers deposited, respectively, on Si and Al were measured on SuperADAM.

On the D17 instrument, the reflectivity profiles were measured using three angles *θ*=0.5°,1°,2° in ToF-mode between λ=2 and 25 Å. The reflected intensity (and the direct beam) in ToF can be measured in energy dispersive mode by acquiring the full neutron wavelength spectrum at once. The reflectivity is calculated as the ratio between the reflected and the direct wavelength spectra. The background, uncorrelated with the instrument timing, was evaluated by looking at a region of the detector far from the specular reflection. This background has been subtracted from the reflected and the direct beam spectra. We repeated the neutron reflectivity measurement on SuperADAM in angle dispersive mode at fixed wavelength to λ=4.4 Å. The sample angle has been changed in order to measure reflectivity for the corresponding *q*.

In addition to neutron reflectivity (*R*), the absorption (*A*) has been measured at the same time. A liquid nitrogen-cooled HPGe-detector has been used to capture the prompt *γ*-ray response of *n*+^10^B reaction

2.1 $$\begin{array}{rl} n+{\;}^{10}B\to {\;}^{7}{\mathrm{Li}}^{*}+\alpha & \to {\;}^{7}\mathrm{Li}+\alpha +\gamma (478\;\mathrm{KeV})+2.31\;\mathrm{MeV}\;(94\%)\\ & \to {\;}^{7}\mathrm{Li}+\alpha +2.79\;\mathrm{MeV}\;(6\%). \end{array}$$

**Figure 3.**
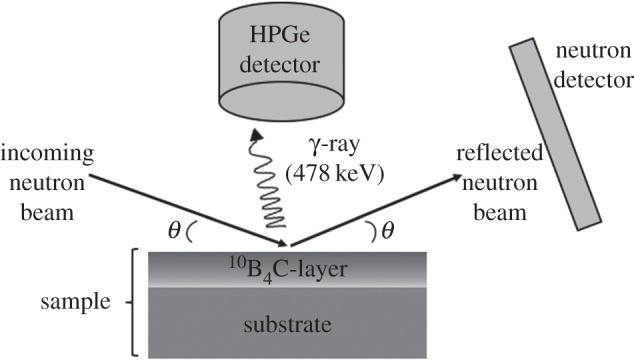
Schematic of the experiment on SuperADAM. A HPGe-detector was used to measure the neutron absorption in the ^10^B_4_C layer exploiting the capture of the prompt *γ*-ray (478 KeV) of the *n*+^10^B reaction.

In the 94% branch, a prompt 478 KeV *γ*-ray is emitted. The schematic representation of the experimental set-up at SuperADAM is depicted in figure 3. Taking into account both the HPGe-detector efficiency and the solid angle, we estimate the absolute efficiency for the 478 KeV *γ*-ray photo-peak detection to be about 5%. The HPGe-detector has been energy calibrated using a ^22^Na source. For a given sample, we record for each angle a spectrum for the HPGe-detector and a neutron detector image of the reflected neutrons. The normalization is given by the direct beam which was also recorded.

Each point of the absorption curve (*A*) has been obtained by fitting the HPGe-detector spectrum, around the 478 KeV *γ*-ray photo-peak, with a model that includes a linear background. The latter is subtracted from the actual number of counts and it takes the Compton background due to other *γ*-ray energies into accounts.

Since the sample length is not infinite there will be a certain point in the angular scan when the size of the beam coincides with the projected size of the sample, this is the so-called over-illumination angle (*θ*_over_). Therefore, the raw intensity of the reflection rises until *θ*_over_ and then behaves as an absolute reflectivity profile. Hence a data correction was applied in order to transform the intensity of the reflection into reflectivity.

The model considered to fit the data have been explained previously, the absorption and the reflected intensities are fitted simultaneously with a least-squares minimization. The scattering length densities (real and imaginary parts), layer roughnesses *σ*_*r*_, layer thickness and HPGe-detector efficiency, are the free fitting parameters. The HPGe-detector efficiency takes the efficiency of the detector for the 478 KeV *γ*-ray photo-peak and the solid angle subtended into account. We model the surface roughness, *σ*_*r*_, at the *i*th interface as an error function on the position of the interface as $e^{\left(-k_{i}\cdot k_{i+1}\cdot \sigma_{r}^{2}\right)}$, where *k*_*i*_ and *k*_*i*+1_ are the normal component of the wavevector of the media *i* and *i*+1 which define the interface.

## 3. Results and discussion

**Figure 4.**
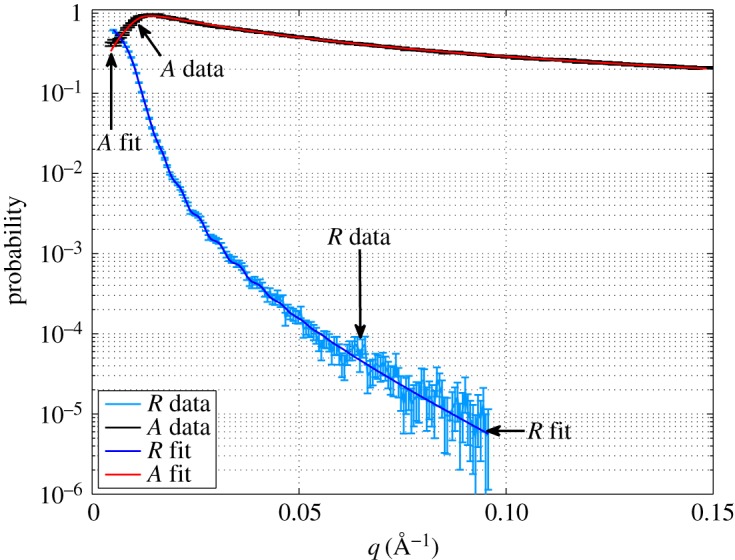
Measured and fitted reflectivity (*R*) and absorption (*A*) probabilities for the 100 nm ^10^B_4_C sample deposited on Si as a function of the momentum transfer (*q*). (Online version in colour.)

Figure 4 shows the reflectivity and absorption profiles for the 100 nm ^10^B_4_C sample deposited on Si. The over-illumination correction is here applied to the data to visualize the absolute reflectivity curve. The sample of 1 μm deposited on Al has also been measured, but no specular reflection has been observed while absorption is comparable to that of the sample deposited on Si. The specular reflection is attenuated by the surface roughness of the film, which is for Al substrates in the order of a few tens of nanometres. The Al substrate is etched and this to explain its large roughness. The high roughness is not a characteristic of Al in general, but it can also be very flat if manufactured in a different way.

Off-specular reflection was not observed in any sample (Si or Al) or it is at or below the background level. This suggests that there is no in-plane structure of the coating. By comparing this result to the AFM measurements, we can conclude that the surface features are randomly scattered over the surface without any correlation, as expected in a sputtering process [20,25].

From table 1, we notice that a thin oxide layer is present on top of the converter layer. The SLDs vary significantly in a few nanometres (table 2). We can choose to fit the data of the 100 nm with a model that includes either two or three layers. In the first case, we consider a unique converter layer deposited on a substrate and in the second case we also add an oxide layer at the air/converter interface. As 1 μm is far beyond the sampling depth of any neutron reflectometer and any reflection from the substrate is attenuated by the ^10^B_4_C layer, for the thicker sample we can exclude the substrate contribution in the model; we can fit the data with either one single layer or two. The adopted choice of model is important and has a significant influence on the interpretation of the result. The oxide layer can be modelled as a rough layer deeply overlapped with the ^10^B_4_C layer below, as specular reflectivity cannot distinguish between a rough or a diffuse interface. We used a smearing function on the one-dimensional scattering potential at each interface.

We fix the SLD of the Si substrate to 2.14×10^−6^ Å^−2^, its roughness is a free fitting parameter. If we exclude the oxide layer the fit converges with a reduced chi-square of ${\tilde{\chi }}^{2}=1.7$, without setting any boundary condition on the parameters of the fit. In table 3, the fitting parameters are listed. We define the error on a parameter as the change of a parameter which produces a change of the reduced ${\tilde{\chi }}^{2}=1$. Note that this method to calculate the errors on the parameters of the fit can, but not necessarily, lead to an overestimation of such errors [30]. More about the errors on the parameters of the fit can be found in the electronic supplementary material where we compare the errors obtained with this method and with a standard covariance matrix method obtained by fitting the data with MINUIT [31] for one of the samples measured.

**Table 3.** Fitting parameters including results of the fit with and without the oxide layer on the sample surface. Values listed refer to ^10^B_4_C deposited onto Si.

sample	model	layer	*d* (nm)	*N*_*b*_ (10^−6^ Å^−2^)	*σ*_*r*_(*nm*)
100 nm	2 layers	1 (^10^B_4_C)	121±2	(2.50±0.04)−(1.11±0.04)*i*	4.2±0.8
		2 (substrate)	—	2.14	5±1
100 nm	3 layers	1 (oxide)	3.9±0.5	(3.52±0.06)−(0.8±0.2)*i*	3.6±0.4
		2 (^10^B_4_C)	115±2	(2.11±0.06)−(1.18±0.05)*i*	4±1
		3 (substrate)	—	2.14	5±1
1 μm	1 layer	1 (^10^B_4_C)	—	(2.48±0.05)−(1.01±0.04)*i*	3.1±0.3
1 μm	2 layers	1 (oxide)	3.5±0.5	(3.15±0.06)−(0.5±0.1)*i*	4.6±0.4
		2 (^10^B_4_C)	—	(2.08±0.05)−(1.10±0.04)*i*	2.1±0.3

In case we include the oxidized layer, the fit may converge to many different local minima because of the number of free fitting parameters; it converges with ${\tilde{\chi }}^{2}=1.1$ only when the parameters are allowed to vary in physically acceptable boundaries based on the XPS measurements: e.g. the SLD is allowed to vary in the ranges given in table 2. Both samples deposited on Si show similar results.

Referring to table 3 in the case the oxide layer is excluded, the resulting *N*_*b*_ is in the range of the value given in table 2, but it does not represent the oxide or the ^10^B_4_C layer, rather it is the mixture of both. The result of the fit is physically correct only when the extra layer is included by the fit which is driven by complementary measurements. The reflectivity curve is produced by the interfaces and absorption curve is instead a volume effect; more precisely, from equation (1.9) we can calculate that the absorption is mainly determined by a few hundreds of nanometres on the surface, while the oxidized layer plays a more significant role in the reflectivity curve. In the absorption curve, the major contribution is represented by the ^10^B_4_C underneath. Hence, if we do not include the oxide interface in the fit, the real part of the one-dimensional potential increases.

The absorption (*A*) is given by the imaginary part of the scattering potential and we can assume that it is entirely determined by the ^10^B content; thus from the imaginary part of *N*_*b*_ the ^10^B number density is univocally determined. With Im(*N*_*b*_)=1.1×10^−6^ Å^−2^, it is $n_{{\;}^{10}B}=0.103\;$ Å^−3^.

The layer roughnesses are compatible to the result of the AFM measurement. The thinner layer thickness is estimated to be (121±2) nm. As the oxidized layer is a diffuse interface into the ^10^B_4_C, we get from the fit a layer with a large relative roughness.

## 4. Reflection of neutrons by converters at grazing angle used in neutron detection

The efficiency of a thermal neutron detector exploiting a solid neutron converter increases rapidly as the angle between the incoming neutrons and the converter decreases below 10°. Detailed analytical calculations are given in [32]. It has also been demonstrated by experimental results [12,15–17]. As already mentioned, the neutron reflection must be avoided in neutron detection because it limits the maximum efficiency that can be attained. Moreover, the detector requirements for low sensitivity to background [6,7] are becoming more and more strict [33–35]. Background events can arise from *γ*-ray detection or background neutrons that can give rise to misaddressed events. The *γ*-ray sensitivity must be kept very low with respect to neutron efficiency, typically 10^−6^ [36]. The same order of magnitude must be kept for the neutron background considering that the neutron detection efficiency is much larger than that of *γ*-rays. The neutrons that are reflected by the converter in a detector can strongly contribute to the background. Therefore, the neutron reflection must be taken into account in the detector concept even if it is very low.

**Figure 5.**
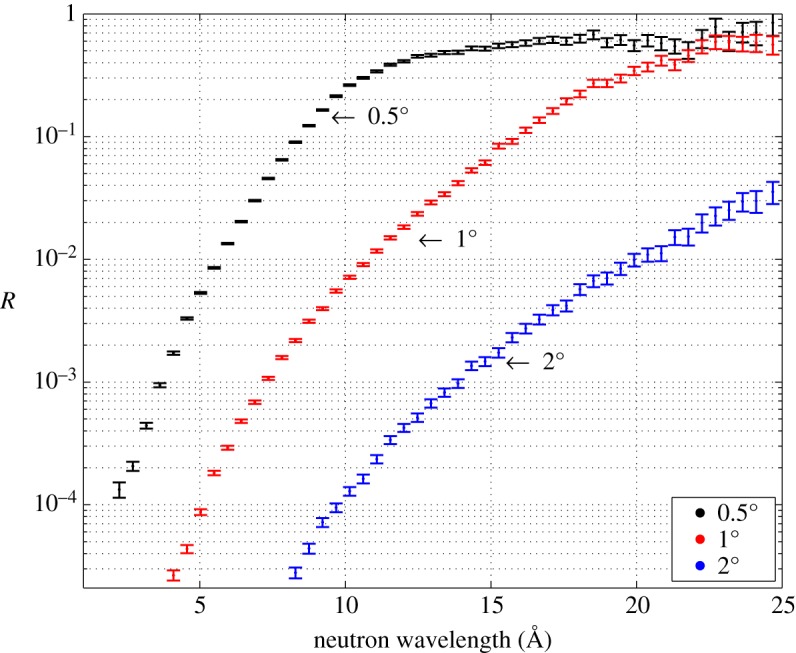
Measured reflectivity of 1 μm^10^B_4_C deposited on Si as a function of the neutron wavelength (λ) for three different angles. (Online version in colour.)

We characterized the ^10^B_4_C layers deposited on Si; the converter roughness on this substrate is a few nanometres due to low surface roughness of the substrate. Figure 5 shows the reflectivity curve for the 1 μm sample on Si as a function of the neutron wavelength (ToF measurement) for three different angles (0.5°, 1° and 2°). Note that for wavelengths larger than 20 Å, if we use a converter inclined at 1° (red curve) about 30% or more of the neutrons are reflected, thus not converted. Already at 2°, the reflection is negligible for most potential applications. Note that the data in figure 5 are presented as raw data recorded at the instrument. Background subtraction has not been applied here (background subtraction was applied to the data plotted as a function of *q* in figure 4) and this can affect the lower intensities.

For the 1 μm thick sample deposited on Al, no specular reflection was observed or the reflected neutron intensity was below the background of the instrument (≈10^−6^) at any value of *q*. The specular reflection is attenuated by the surface roughness. In order to diminish the reflection effect in a detector, it is sufficient to have a rough surface, as for Al where it is a few tens of nanometres. This can be of importance for detectors based on micro-strips and solid converters. Operated at small angle, the absorber deposition on glass could not have a large enough roughness to avoid significant reflection. It has to be pointed out that excessive roughness will also degrade the efficiency at small angles [12,37]. When the roughness becomes comparable to the neutron capture fragments path lengths in the converter (≈1 μm for ^10^B_4_C) the surface cannot be considered flat anymore. The flatness is essential for the neutron to traverse a large thickness of ^10^B, and the conversion fragments to be close to the surface to be able to escape. If the roughness starts to be comparable to the conversion fragments ranges, this assumption is not valid anymore and it might results in a drop in the expected efficiency.

## 5. Conclusion

A new technique to measure and exploit the neutron reflectivity along with the neutron-induced fluorescence for layers of strongly neutron absorbing materials has been described. The theoretical model has been developed for single and multiple layers, and it has been understood in the light of the measurements performed on ^10^B_4_C films deposited on both Si and Al. Moreover, theory and experiment have verified that reflectivity profiles, even on highly absorbing films, can be measured in ToF or in monochromatic mode without affecting the results (if we can assume constant imaginary SLD, meaning there are no absorption resonances).

The understanding of the partially oxidized surface layer is crucial to obtain a fit with reasonable physical constraints. Film thickness, SLDs and surface roughnesses can be determined from the fit. We extended the neutron-induced fluorescence technique to measure and fit the absorption over a wide *q*-range not only limited below the critical edge. Neutron reflectometry measured together with fluorescence is a powerful non-destructive tool to directly obtain the number density of the absorbing isotope, ^10^B in our experiment.

We characterized the ^10^B_4_C layers in order to understand the amount of reflection that must be minimized for application in neutron detection. Surface roughness helps to attenuate the reflection as it was observed on the film deposited on Al. At a very grazing angle (≈1°) the reflection does not only reduce the maximum detection efficiency that can be attained but can also generate a source of background that must be taken in into account in the detector concept.

Finally, we note that the methods and theory developed open up the ability of using neutron reflection as a diagnostic technique on highly absorbing films.

## Supplementary Material

Neutron induced-fluorescence reflectometry on 10B4C data

## Data accessibility

The raw reflectometry data (doi:10.6084/m9.figshare.2055993.v1) of the two experiments described in this manuscript can be downloaded from https://figshare.com/articles/Neutron_induced_fluorescence_reflectometry_on_10B4C_data/2055993. Information about the preliminary characterization of the samples can be found in [20,25].

## References

[1] Pike R, Sabatier P 2002 *Scattering—scattering and inverse scattering in Pure and Applied Science*, vol. 2, pp. 1198–1208. London, UK: Academic Press.

[2] Fermi E, Zinn WH 1946 *Reflection of neutrons on mirrors*. Los Alamos National Laboratory, U.S. Atomic Energy Commission. Oakridge, TN: Manhattan District.

[3] Aksenov VL, Nikitenko YV, Radu F, Gledenov YM, Sedyshev PV 2000 Observation of resonance enhanced neutron standing waves through (*n*,*α*) reaction. *Physica B* 276–278, 946–947. (doi:10.1016/S0921-4526(99)01270-3)

[4] Zhang H, Gallagher PD, Satija SK, Lindstrom RM, Paul RL, Russell TP, Lambooy P, Kramer EJ 1994 Grazing incidence prompt gamma emissions and resonance-enhanced neutron standing waves in a thin film. *Phys. Rev. Lett.* 72, 3044–3047. (doi:10.1103/PhysRevLett.72.3044) 10056053 10.1103/PhysRevLett.72.3044

[5] Schneck E, Jentschel M, Gege C, Tanaka M, Deme B 2013 Grazing-incidence neutron-induced fluorescence probes density profiles of labeled molecules at solid/liquid interfaces. *Langmuir* 29, 4084–4091. (doi:10.1021/la400162y) 23461763 10.1021/la400162y

[6] Peggs S *et al.* 2013 *ESS Technical Design Report*, ESS-doc-274.

[7] Kirstein O *et al.* 2014 *Neutron Position Sensitive Detectors for the ESS*. In *Proc. of the 23rd Int. Workshop on Vertex Detectors, 15–19 September 2014, Macha Lake, The Czech Republic*, PoS(Vertex2014)029 (http://arxiv:1411.6194).

[8] Cho A 2009 Helium-3 shortage could put freeze on low-temperature research. *Science* 326, 778–779. (doi:10.1126/science.326_778) 19892947 10.1126/science.326_778

[9] Zeitelhack K 2010 Search for alternative techniques to helium-3 based detectors for neutron scattering applications. *Neutron News* 23, 10–13. (doi:10.1080/10448632.2012.725325)

[10] Dianoux A-J, Lander G 2013 *Neutron data booklet*, 2nd edn. Philadelphia, PA: OCP Science.

[11] Buffet JC, Correa J, Van Esch P, Guerard B, Khaplanov A, Piscitelli F 2012 Study of a 10*B*-based Multi-Blade detector for neutron scattering science. In *Conf. record of Nuclear Science Symp. and Medical Imaging Conf. (NSS/MIC) Anaheim*. *IEEE Trans. Nucl. Sci., Anaheim, CA, 27 October–3 November*, pp. 171–175. Piscataway, NJ: IEEE.

[12] Piscitelli F, Buffet JC, Clergeau JF, Cuccaro S, Guerard B, Khaplanov A, La Manna Q, Rigal JM, Van Esch P 2014 Study of a high spatial resolution 10B-based thermal neutron detector for application in neutron reflectometry: the Multi-Blade prototype. *J. Instrum.* 9, P03007 (doi:10.1088/1748-0221/9/03/P03007)

[13] Piscitelli F 2014 Boron-10 layers, neutron reflectometry and thermal neutron gaseous detectors. PhD thesis, Institut Laue-Langevin, Grenoble, France, and University of Perugia, Perugia, Italy. (http://arxiv:1406.3133).

[14] Piscitelli F 2015 Novel boron-10-based detectors for neutron scattering science. *Eur. Phys. J. Plus* 130, 27 (doi:10.1140/epjp/i2015-15027-3)

[15] Henske M, Klein M, Köhli M, Lennert P, Modzel G, Schmidt CJ, Schmidt U 2012 The 10B based Jalousie neutron detector—an alternative for 3He filled position sensitive counter tubes. *Nucl. Instrum. Meth. A* 686, 151–155. (doi:10.1016/j.nima.2012.05.075)

[16] Modzel G et al. 2014 Absolute efficiency measurements with the 10B based Jalousie detectors. *Nucl. Instrum. Meth. A* 743, 90–95. (doi:10.1016/j.nima.2014.01.007)

[17] Nowak G et al. 2015 Boron carbide coatings for neutron detection probed by x-rays, ions, and neutrons to determine thin film quality. *J. Appl. Phys.* 117, 034901 (doi:10.1063/1.4905716)

[18] van Vuure TL et al. 2010 First measurements of the inclined boron layer thermal-neutron detector for reflectometry. *IEEE Trans. Nucl. Sci.* 57, 323–327. (doi:10.1109/TNS.2009.2036913)

[19] Croci G et al. 2014 Diffraction measurements with a boron-based GEM neutron detector. *Europhys. Lett.* 107, 12001 (doi:10.1209/0295-5075/107/12001)

[20] Höglund C et al. 2012 *B*_4_*C* thin films for neutron detection. *J. Appl. Phys.* 111, 10490-8 (doi:10.1063/1.4718573)

[21] Sears VF 1992 Neutron scattering lengths and cross sections—special feature. *Neutron News* 3, 29–37. (doi:10.1080/10448639208218770)

[22] Hayter JB, Mook HA 1989 Discrete thin-film multilayer design for X-ray and neutron supermirrors. *J. Appl. Crystallogr.* 22, 35–41. (doi:10.1107/S0021889888010003)

[23] Schiff LI 1955 *Quantum mechanics*, 3rd edn New York, NY: McGraw-Hill, Inc.

[24] Parratt LG 1954 Surface studies of solids by total reflection of X-rays. *Phys. Rev.* 95, 359 (doi:10.1103/PhysRev.95.359)

[25] Höglund C 2010 Growth and phase stability studies of epitaxial Sc-Al-N and Ti-Al-N thin films. PhD thesis, Linköping University, Institut of Technology, Linköping, Sweden.

[26] Höglund C, Zeitelhack K, Kudejova P, Jensen J, Greczynski G, Lu J, Hultman L, Birch J, Hall-Wilton R 2015 Stability of ^10^B_4_C thin films under neutron radiation. *Radiat. Phys. Chem.* 113, 14–19. (doi:10.1016/j.radphyschem.2015.04.006)

[27] Birch J et al. 2013 ^10^B_4_C Multi-Grid as an Alternative to ^3^H*e* for large area neutron detectors. *IEEE Trans. Nucl. Sci.* 60, 871–878. (doi:10.1109/TNS.2012.2227798)

[28] Cubitt R, Fragneto G 2002 D17: the new reflectometer at the ILL. *Appl. Phys. A* 74, s329–s331. (doi:10.1007/s003390201611)

[29] Devishvili A, Zhernenkov K, Dennison AJ, Toperverg BP, Wolff M, Hjörvarsson B, Zabel H 2013 SuperADAM: upgraded polarized neutron reflectometer at the Institut Laue-Langevin. *Rev. Sci. Instrum.* 84, 025112 (doi:10.1063/1.4790717) 23464256 10.1063/1.4790717

[30] Press WH, Flannery BP, Teukolsky SA, Vetterling WT 1988–1992 *Numerical recipes in C: the art of scientific computing*, pp. 689–706. Cambridge, UK: Cambridge University Press.

[31] James F, Roos M 1975 Minuit—a system for function minimization and analysis of the parameter errors and correlations. *Comput. Phys. Commun.* 10, 343–367. (doi:10.1016/0010-4655(75)90039-9)

[32] Piscitelli F, van Esch P 2013 Analytical modeling of thin film neutron converters and its application to thermal neutron gas detectors. *J. Instrum.* 8, P04020 (doi:10.1088/1748-0221/8/04/P04020)

[33] Stahn J, Filges U, Panzner T 2012 Focusing specular neutron reflectometry for small samples. *Eur. Phys. J. Appl. Phys.* 58, 11001 (doi:10.1051/epjap/2012110295)

[34] Stahn J 2014 *ESTIA: A Truly Focusing Reflectometer*, ESS instrument proposal.

[35] Wacklin H 2014 *FREIA: Reflectometer concept for fast kinetics at ESS*, ESS instrument proposal.

[36] Khaplanov A, Piscitelli F, Buffet JC, Clergeau JF, Correa J, van Esch P, Ferraton M, Guerard B, Hall-Wilton R 2013 Investigation of gamma-ray sensitivity of neutron detectors based on thin converter films. *J. Instrum.* 8, P10025 (doi:10.1088/1748-0221/8/10/P10025)

[37] Stefanescu I, Abdullahi Y, Birch J, Defendi I, Hall-Wilton R, Höglund C, Hultman L, Seiler D, Zeitelhack K 2013 Development of a novel macrostructured cathode for large-area neutron detectors based on the ^10^B-containing solid converter. *Nucl. Instrum. Meth. A* 727, 109–125. (doi:10.1016/j.nima.2013.06.003)

